# Porcine Induced Pluripotent Stem Cells Require LIF and Maintain Their Developmental Potential in Early Stage of Embryos

**DOI:** 10.1371/journal.pone.0051778

**Published:** 2012-12-14

**Authors:** De Cheng, Yanjie Guo, Zhenzhen Li, Yajun Liu, Xing Gao, Yi Gao, Xiang Cheng, Junhe Hu, Huayan Wang

**Affiliations:** Department of Animal Biotechnology, College of Veterinary Medicine, Northwest A&F University, Yangling, Shaanxi, People’s Republic of China; Wellcome Trust Centre for Stem Cell Research, United Kingdom

## Abstract

Porcine induced pluripotent stem (piPS) cell lines have been generated recently by using a cocktail of defined transcription factors, however, the features of authentic piPS cells have not been agreed upon and most of published iPS clones did not meet the stringent requirements of pluripotency. Here, we report the generation of piPS cells from fibroblasts using retrovirus carrying four mouse transcription factors (mOct4, mSox2, mKlf4 and mc-Myc, 4F). Multiple LIF-dependent piPS cell lines were generated and these cells showed the morphology similar to mouse embryonic stem cells and other pluripotent stem cells. In addition to the routine characterization, piPS cells were injected into porcine pre-compacted embryos to generate chimera embryos and nuclear transfer (NT) embryos. The results showed that piPS cells retain the ability to integrate into inner and outer layers of the blastocysts, and support the NT embryos development to blastocysts. The generations of chimera embryos and NT embryos derived from piPS clones are a practical means to determine the quality of iPS cells ex vivo.

## Introduction

The first recognized embryonic stem (ES) cell lines were established from the inner cell mass (ICM) of preimplantation blastocysts of 129 strain mice [1], [2]. However, it took nearly two decades to isolate human ES cells from in vitro fertilization (IVF) blastocysts [3]. ES cells cultured in vitro provided powerful cell resources for developmental research and clinical application. So far, the establishment of authentic ES cells was only succeeded in rodents, including mouse and rat [1]–[4]. The domestic pig (Sus scrofa domesticus) is one of the most common livestocks around the world, and also is an ideal animal model for the regenerative medicine due to its close resemblance to human on body size, physical structure and metabolism. Despite the fact that intense efforts have been taken to establish porcine ES cells since the 1990s, few validated successes have been achieved except for the recent establishment of LIF-dependent pig pluripotent stem cells [5] and pig epiblast stem cells (EpiSCs) [6]. Therefore, the application of pigs for the genetic engineering and production of transgenic pigs are highly limited.

Through ectopic expression of defined transcription factors, the mouse somatic cells can be reprogrammed into pluripotent state which shares similar morphology and characteristics with mouse ES cells [7]. Until recently, numerous iPS cell lines have been successfully established in many species, including human [8], [9], rhesus monkey [10], rat [11], pig [12]–[14], sheep [15], canine [16], [17], rabbit [18], goat [19] and bovine [20]. It is anticipated that the establishment of piPS cells can provide in-depth view on the properties of porcine pluripotent stem cells. Whereas, controversy still exists on the morphology, the expression of pluripotent markers and suitable culture conditions for the maintenance of piPS cells (Table S1), most of these reported cell lines morphologically resembled human ES cells and mouse EpiSCs [21], [22]. Notably, retroviral transgenes were not efficiently silenced under current culture conditions [12], [13], [23]. Moreover, only few live-born offspring of germline transmission chimera from iPS cells have been reported [24]–[26], suggesting that these reported reprogrammed porcine iPS cells may only partially reprogrammed and do not process full pluripotent potentials. The difficulty for these iPS cells to produce the cloned animals and viable chimeras might result from the known defects of iPS cells, such as the abnormalities of chromosome and aberrant silencing of Dlk1-Dio3 imprinted domain that arisen during cellular reprogramming. These defects are also likely to impair the application of piPS cells for the regenerative medicine and transgenic animal research [27]–[29]. Moreover, the persistent expression of retroviral reprogramming genes may also disturb the expression pattern of downstream genes in iPS cells [26].

In this study, piPS cells were generated using a combination of four mouse factors (mOct4, mSox2, mKlf4 and mc-Myc, 4F). Unlike previous reports [12], [14], these cells exhibited three-dimensional and tightly packed colonies, similar to mouse and rat ES cells in morphology [1], [4], and were dependent on LIF/STAT3 signaling pathway. In addition to their potentials of self-renewal and pluripotency in vivo and in vitro, the obtained piPS cells were capable of producing chimeric and reconstructed nuclear transfer (NT) embryos. The resulting embryos could develop into the blastocysts. Thus, these piPS cells were qualified to be used as donor cells for development of chimeric or NT pig offspring.

## Materials and Methods

### Cells Culture

The piPS cells established in this study were cultured in piPS medium, including Knock-out DMEM (KO-DMEM, Invitrogen) supplemented with 20% FBS (HyClone), 0.1 mM non-essential amino acids (NEAA, Invitrogen), 1 mM L-glutamine, 10 ng LIF (Millipore, ESG1106), 10 ng bFGF (Millipore, GF003), 0.1 mM β-mercaptoethanol and 50 units/50 mg/ml penicillin/streptomycin, at 38.5°C, 5% CO_2_ in a humidified atmosphere. The piPS cells were maintained on mitotically inactive mouse embryonic fibroblasts (MEFs) feeder layers derived from ICR mice, and passaged using 1 mg/ml Collagenase type IV (Invitrogen) and 0.05% Trypsin (Invitrogen) every 2 to 3 days. Phoenix-A cells, a second-generation retrovirus producer line, were cultured in DMEM with 10% FBS.

The pig specimens were purchased from a licensed local slaughterhouse, Shaanxi Wansheng meat food processing Co., Ltd., a branch of YURUN Group. And they permitted the porcine parts to be used for the scientific and educational research in our lab. The treatment of porcine samples was based on the protocol of the farm animal research guidelines approved by the Animal Research Committee of Northwest A&F University. The porcine embryonic fibroblasts (PEF) were prepared from a 35-day-old porcine fetus. After washing with PBS plus 100 units/ml penicillin and 100 mg/ml streptomycin, the boneless tissues were cut into pieces, and digested by 3–5 ml of collagenase IV (1 mg/ml) at 38.5°C for 5–6 hours. Following the addition of equal amount of culture medium into the digestion, the mixture was centrifuged at 200 g for 5 min at room temperature. The supernatant was removed and cell pellet was resuspended in DMEM with 15% FBS, and 1×10^6^ cells were seeded on a 100 mm dish at 38.5°C, 5% CO_2_ for 72 hours. Cells grown to 90% confluence were collected and cryopreserved for future use.

To perform the live-cell imaging experiments, piPS cells were treated with 0.05% Trypsin for 5 min, and then seeded on a 35 mm culture plate with MEF feeder at 38.5°C, 5% CO_2_ for 24 hours, images of cultured cells were recorded in 2 min intervals by Leica microscope (Leica AF6000, Germany).

### Retroviral Production and Generation of Porcine Induced Pluripotent Stem Cell Lines

The day before transfection, Phoenix-A cells were planted on a 6-well plate with 2×10^6^ cells per well. After 24 hours, pMXs plasmids carrying mouse Oct4, Sox2, Klf4 and c-Myc were transfected into Phoenix-A cells using Lipofectamine 2000 (Invitrogen) according to the manufacturer’s recommendations. 48 hours after transduction, the medium with virus particles was collected and filtered through 0.45 mm cellulose acetate filter (Millipore). The equal amounts of supernatants containing each of the four factors was mixed and used to infect PEF cells in the presence 4 µg/ml Polybrene for 12 hours. To increase infection efficiency, a second infection with fresh virus was conducted. After infection, PEF cells were collected and counted. The infected cells were re-plated on 60 mm dish (50,000 cells per plate) with iPS medium. To optimize the culture conditions, the following three types of iPS media were tested, M1 (DMEM/F12 supplemented with 20% Knock-out Serum Replacement (KSR, Invitrogen), 0.1 mM NEAA, 1 mM L-Glu, 0.1 mM β-Me and 10 ng/ml bFGF), M2 (KO-DMEM supplemented with 20% FBS, 0.1 mM NEAA, 1 mM L-Glu, 0.1 mM β-Me, 10 ng/ml LIF and 10 ng/ml bFGF) and M3 (KO-DMEM supplemented with 20% FBS, 0.1 mM NEAA, 1 mM L-Glu, 0.1 mM β-Me, 10 ng/ml LIF, 10 ng/ml bFGF and 1 mM Valproic acid, VPA ). Small ES-like colonies appeared 6–8 days post-infection. After another 3–5 days, the colonies became big enough to pick up. Then the fine colonies were picked up and digested with 0.05% Trysin for 5 min, and transferred into a 96-well plate with MEF feeder. 3–5 days later, the colonies in 96-well plate re-appeared and were passaged as routine procedure for mouse ES cells culture. To monitor the infection efficiency and silencing of exogenous gene expression during PEF reprogramming, the pMXs-GFP was mixed into virus particles of four factors.

The media lacking either LIF or bFGF and addition of JAK I inhibitor (Calbiochem #420099, 3 µM) were also used to culture piPS cells to test the influence of LIF signal pathway on piPS cell self-renewal. For 5 days growth, cells were harvested and used to detect the phosphorylation of STAT3 and the expression of pluripotent genes.

### Alkaline Phosphatase Staining and Karyotype Analysis of piPS Cells

The alkaline phosphatase (AP) activity of piPS cells was determined by AST Fast Red TR and α-Naphthol AS-MX Phosphate (Sigma Aldrich) according to the manufacturer’s instructions. Briefly, cells were washed twice using ice-cold PBS, fixed with 4% paraformaldehyde in PBS (pH 7.4) for 15 min at room temperature, followed by washing three times using ice-cold PBS. Cells were then incubated at room temperature (RT) in the solution containing Fast Red TR 1.0 mg/ml, Naphthol AS-MX 0.4 mg/ml in 0.1 M Tris Buffer. After 10–20 min incubation, the AP positive iPS colonies showed in red color.

Karyotype analysis was performed following the standard procedure described in Cell Biology Laboratory Manual (http://homepages.gac.edu/~cellab/chpts/chpt10/ex10-6.html).

### Immunocytochemistry

For immunocytochemical analysis, cells were fixed with 4% paraformaldehyde in PBS (pH 7.4) for 15 min at RT. Fixed cells were washed twice with ice-cold PBS, incubated with PBS containing 0.1% Triton X-100 for 10 min, and washed three times with PBS. After blocking in BSA-blotting buffer (1% BSA, 0.1% Tween 20 in PBS) for 30 min, cells were incubated in BSA-blotting buffer containing primary antibodies [anti-Nanog (ab80892, 1∶200, Abcam), anti-SSEA1 (90230, 1∶50, Millipore), anti-SSEA4 (90231, 1∶50, Millipore), anti-TRA-1-60 (90232, 1∶50, Millipore), anti-TRA-1-81(90233, 1∶50, Millipore)] in a humidified chamber for 1 hour at 37°C or overnight at 4°C. The cells were washed and stained for 1 hour in secondary antibodies [anti-mouse (ab6787, 1∶200, Abcam) or anti-rabbit (HG010, 1∶200, DingGuo, China) secondary antibody conjugated with FITC or TRITC]. For nuclear staining, fixed cells were incubated for 1–2 min with 10 µg/ml fluorescent dye of Hochest33342 (Hoe). Microscopy was performed on a Leica fluorescence microscope.

### Flow Cytometry Analysis

The surface antigen SSEA4 and exogenous GFP in reprogrammed PEF cells were examined by flow cytometry. Cells were trypsined and fixed in 4% paraformaldehyde in PBS (pH 7.4) for 30 min at room temperature, and followed by washing three times using washing buffer (PBS with 0.1% Tween-20). After blocked in PBS with 1% BSA for 30 min at room temperature, cells were incubated in PBS containing 0.1% tween-20 and SSEA4 primary antibody (90231, 1∶50, Millipore) overnight at 4°C. Goat anti-mouse IgG conjugated with Cy3 (CW0145, CWBIO, China) were applied for one hour at room temperature. After washing three times, cells were then analyzed by the flow cytometer (Beckman Coulter).

### Reverse Transcription Polymerase Chain Reactions

Total cellular RNAs of porcine cells were extracted by TRIzol Reagent (Invitrogen) according to the manufacturer’s procedure. The RNA samples were examined by the measurement of OD260/280 ratio and the samples with a ratio of 2.0 were used for reverse transcription. The DNase I treatment was utilized in RNA samples to remove contaminating genomic DNA. One microgram RNA was reverse transcribed using oligo-dT primer and RevertAid™ reverse transcriptase (Fermentas, Canada). PCR reactions were performed for 30–35 cycles at 94°C 30 sec, 54–60°C 30 sec, and 72°C 30 sec. PCR products were analyzed in 1.5% agarose gel with ethidium bromide. Non-template negative controls (RT-) were also performed to monitor non-specific reactions, and β-Actin or GAPDH was used as internal controls. Quantitative RT-PCR analyses were performed in triplicates using SYBR Green PCR Master Mix (Takara) and detected with LineGene 9600 Real-Time PCR System software (BIOER), and data were normalized to those of β-Actin or GAPDH. Primer sequences are provided in Table S2.

### Bisulfite Genomic Sequencing

Bisulfite treatment was performed using the CpGenome modification kit (QIAGEN) according to the manufacturer’s recommendations. Treated DNAs were PCR amplified using primers (Oct4-Me) (Table S2). Amplified products were cloned into pGEM-T Easy vector (Promega). Six randomly selected clones from each gene were sequenced with the T7 forward and SP6 reverse primers.

### EB Formation and Spontaneous Differentiation

The suspension culture of porcine iPS cells was grown in a 35 mm Petri dish (2 ×10^6^ cells/plate) in iPS medium without bFGF or mLIF. The culture medium was replaced every two days. After the 5-day suspension culture, EBs were re-plated in a gelatin coated culture plate to differentiate, and then cell samples were collected at day 5 and day 10. Total RNAs were isolated and the realtime RT-PCR assay was conducted to detect the markers of three germ layers, including *NESTIN* for ectoderm, *DES* for mesoderm and *NCSTN* for endoderm. Meanwhile, 1 µM retinoic acid (RA) was added in iPS medium without bFGF and mLIF to induce the differentiation. Cells after differentiation for 5 days and 10 days were harvested for detection of the markers of differentiation.

### Teratoma Formation

The usage of live adult mice in this study was approved by Institutional Animal Care and Use Committee, which was subjected Experiment Manage Committee of Northwest A&F University. The animal breeding and tissue sections were conducted in the Department of Laboratory Animal Science of Peking University Health Science Center, which has the licensed animal research facility to provide these commercial services. piPS cells (3–5×10^6^ cells per injection) were injected subcutaneously into CB-17 SCID mice. Three porcine iPS cell lines were used to generate teratoma and six mice were injected for each cell lines. In 6–9 weeks, two tumors from cell PS23 and one tumor from cell PS24 were obtained. Tumors were processed for hematoxylin–eosin staining.

### Microarray Analysis

Transcriptional profiles of PS24, 30AC5 and PEF were analyzed to evaluate the reprogramming of porcine iPS. Total RNAs were extracted by RNeasy Mini Kit (QIAGEN),labeled using IVT Labeling Kit, (Affymetrix), hybridyed to GeneChip (Affymetrix GeneChip Porcine Genome Array, 23937 probes), scanned in GeneChip Scanner 3000 (Affymetrix), and quantified by CapitalBio Corporation (Beijing, China). The data were analyzed with GeneSpring GX 11.0 analysis software (Agilent Technologies, Inc.). Datasets from GEO were imported into GeneSpring GX using a guided workflow step to identify the interested targets. Robust Multi-chip Averaging (RMA) algorithm was used for summarization of Affymetrix expression data. The data were then filtered based on their flag values P (present) and A (absent) to remove probe sets for which the signal intensities for all the treatment groups in the lowest 20 percent of all intensity values (20640 probes left). Hierarchical cluster analysis was performed to assess correlations among samples for each identified gene set with Euclidean distance and complete linkage statistical methods.

### Western Blotting

Cells were washed with ice-cold PBS and lysed at 4°C for 30 min in 10 mM Tris-HCl (pH 7.5), 150 mM NaCl, 0.5% Nonidet P-40, 1 mM PMSF and other protease inhibitors cocktail. The lysate was centrifuged with 13,000 rpm at 4°C for 15 min, and transferred the supernatant into a new 1.5 mL Eppendorf tube. The protein samples were mixed with 4× SDS-PAGE loading buffer (200 mM Tris-HCl, pH 6.8, 8% SDS, 0.2% Bromophenol blue, 40% Glycerine, 8% β-Me), and heated in 95°C for 5 min,, and then subjected to SDS-PAGE. After the electrophoresis, proteins were transferred to a Nitrocellulose (NC) membrane for 45 min at 20 V by the semi-dry electrophoretic transfer. The membrane was blocked by the blocking buffer (20 mM Tris-HCl, pH 7.6, containing 137 mM NaCl, 0.1% Tween 20 and 8% dried nonfat milk) for 1 h at 37°C, and then incubation overnight at 4°C with primary antibody (Tyr705) against phosphorylated-STAT3 (1∶500, SAB) and GAPDH (1∶1000; Beyotime, China). After washing with PBS-T buffer (20 mM Tris-HCl, pH 7.6, 137 mM NaCl, 0.1% Tween 20) for three times, the membrane was then incubated with HRP-conjugated second antibody (1∶1000; Beyotime, China) for 1 hour at 37°C. Rinsed the membrane in TBS-T three times for 5 min each at room temperature and performed ECL (Pierce) and detected with Chemiluminescent Imaging System (Tanon-4200, China).

### Generation of Chimera Embryos from piPS Cells

Porcine oocytes were collected from ovarian follicles, and cultured at 38.5°C for 42 hours to allow the emission of first polar body in M199 medium supplemented with 3.05 mM D-glucose, 0.91 mM Na-Pyruvate, 10 UI/ml hCG, 2.5 UI/ml FSH, 10 UI/ml PMSG, 0.57 mM Cysteine, 10 ng/ml EGF, 10 µg/ml ITS and 50 units/50 mg/ml penicillin/streptomycin. The prepared oocytes were subjected to generate parthenogenetic embryos in vitro. First, electric activation was performed with electric pulse using electrofusion apparatus (Cyto-pause 4000, Cyto-pause Sciences) in Cytofusion medium (Cyto-pause Sciences). After rinsing in M199 medium, oocytes were transferred into 2 mM 6-dimethylamino-purine (Sigma-Aldrich) for an additional 2 h, and then were transferred into PZM-3 medium [30]. After 3 days of incubation, 4–8 cell stage embryos were selected for the injection of piPS cells. About 10 piPS cells were injected into each embryo by micromanipulation. The injected embryos were then transferred into PZM medium for another 3–4 days. PEFs-RFP injected or without any cells injected embryos were used as controls. The number of blastocysts and chimera blastocysts were counted and in vivo embryos was collected from sows in 3.5 day after natural mating and cultured and treated similarly to a forementioned for chimera detection.

### Nuclear Transfer of Porcine Embryos

The procedure of nuclear transfer was based on the previous reports [30]–[32]. The oocytes were collected and matured as in chimera embryos generation. The in vitro maturation (IVM) oocytes were enucleated by removing the first polar body along with adjacent cytoplasm containing the metaphase plate using a micropipette, followed by injection of a porcine iPS cell. The couplets were then washed once and incubated in M199 with 10% FBS at 38.5°C in humidified air containing 5% CO_2_ for at least 30 min. Cell fusion was conducted in Cytofusion medium using the electrofusion apparatus. After the fusion, oocytes were cultured in PZM3 medium for 3 days and the embryos were transferred into new droplets of PZM-3 medium for another 4 days to process the embryonic development.

### Statistical Analysis

Values were presented as the mean±SD. Statistical significance was assessed by using Student’s *t* test where indicated in the figure legends.

## Results

### Generation of Porcine Induced Pluripotent Stem Cells

**Figure 1:**
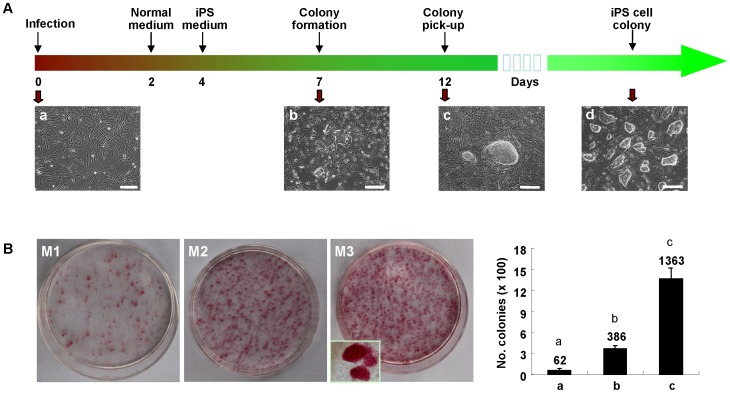
Generation of piPS cells with mouse four defined factors (4F) by retrovirus transduction. A, The scheme of reprogramming porcine fibroblasts into piPS cells. (a) The PEF (less than 3 passages) was infected by 4F; (b) At 6–8 days post-infection, cells formed the small clusters; (c) At 10–14 days post-infection, the large colonies can be picked up and transferred to a new plate with MEF feeder; (d) The piPS clones in the 20^th^ passage. Scale bars, 200 µm. B, Three culture media were used to determine the reprogramming efficiency (50,000 cells/60 mm dish) for 12 days. The inset shows the enlarged AP positive colonies. AP positive colonies in each 60-mm dish were counted and plotted in the right panel. Data indicate mean ± SD (n = 3). Different letters (a, b, c) indicate significantly different between two groups, *p*<0.01 by Student’s *t* test.

A schematic diagram of the generation of porcine iPS cells is summarized in Figure 1A. Amphotropic retrovirus containing four mouse reprogramming factors (mOSKM) were transfected into porcine embryonic fibroblasts (Fig. 1A, a). Infection efficiency of retrovirus was estimated using a control retrovirus expressing GFP (Fig. S1A). Small colonies could be observed at 7–8 days after infection (Fig. 1A, b). These colonies were picked up on days 12–14 and transferred to a fresh plate with MEF feeders (Fig. 1A, c). Within 3–4 passages the typical iPS cell clones could be observed and continuously cultured for over 20 passages (Fig. 1A, d). To determine the optimal culture condition, the infected PEF cells (50,000 cells/60 mm dish) were cultured in three different media (M1, KSR+bFGF; M2, FBS+LIF+bFGF and M3, FBS+LIF+bFGF+VPA). Ten to twelve days after infection, cells were stained by alkaline phosphatase (AP) assay to detect the cell reprogramming efficiency (Fig. 1B). The result of AP assay revealed that the clone formation rate (0.77%) in M2 medium was clearly higher than that in M1 medium (0.12%). Also, the addition of histone deacetylase inhibitor VPA further increased the efficiency of cell reprogramming (2.7%) (Fig. 1B).

**Figure 2:**
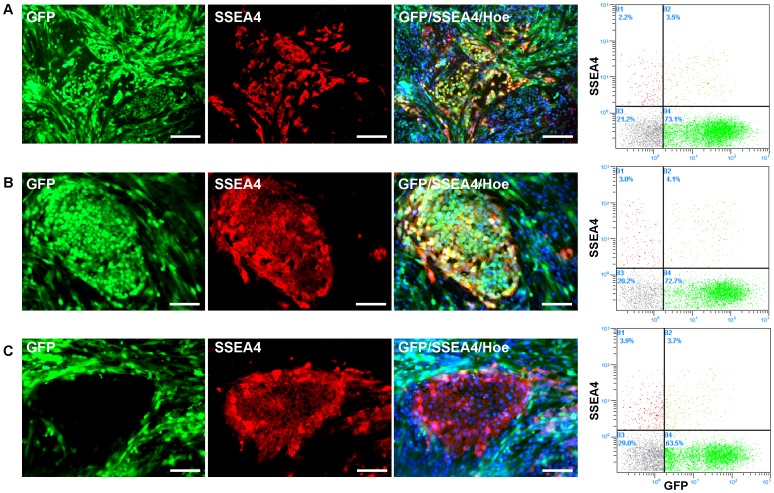
Expression of SSEA4 during the process of PEF reprogramming. The pMXs-GFP was co-transfected with mouse 4F into PEF cells. The immunofluorescence assay of SSEA4 was conducted in different days, and the cell populations were sorted by the flow cytometer (right panel). A, At 8 days post-infection; B, At 12 days post-infection; C, At 16 days post-infection. Hoe, Hoechst 33342. Scale bars, 200 µm for a–b; 100 µm for c.

To further investigate the progress of cell reprogramming, retroviral pMXs-GFP was co-transfected with 4F to monitor the expression of exogenous genes in infected PEFs. In the early stages of reprogramming, the small colonies retained the expression of GFP (Fig. S1B, a–b). Ten to twelve days after infection, the GFP fluorescence in many colonies was absent (Fig. S1B, c–d). Some of these clones continuously grew and displayed three-dimensional morphology (Fig. S1B, e–f). The results of semi-quantitative RT-PCR analysis confirmed the down regulation of expression of transgenes (mOSKM) and GFP in two clones (iF1 and iF2), although the silencing was incomplete (Fig. S1C). In addition, some flat colonies absent GFP fluorescence could be observed when the primary induced cells were continuously cultured for over 16 days, but these flat cell colonies were prone to differentiate in M2 medium and difficult to be passaged (Data was not shown). The expression of cell surface marker SSEA4 was detected by the immunofluorescence and flow cytometry during various days after infection, showing that SSEA4 was expressed exclusively in reprogrammed cells (Fig. 2). The results showed that SSEA4^+^ cells increased from 5.7% at 8 days post-infection to 7.6% at 16 days post-infection and at the same time, the percentage of SSEA4^+^/GFP^-^ cells nearly doubled from 2.2% to 3.9% (Fig. 2, right panel). These data revealed that reprogramming process, which indicated by the expression of SSEA4 and silencing of retroviral GFP, lasted more than 10 days in our experimental conditions.

### Characterization of piPS Cells

**Figure 3:**
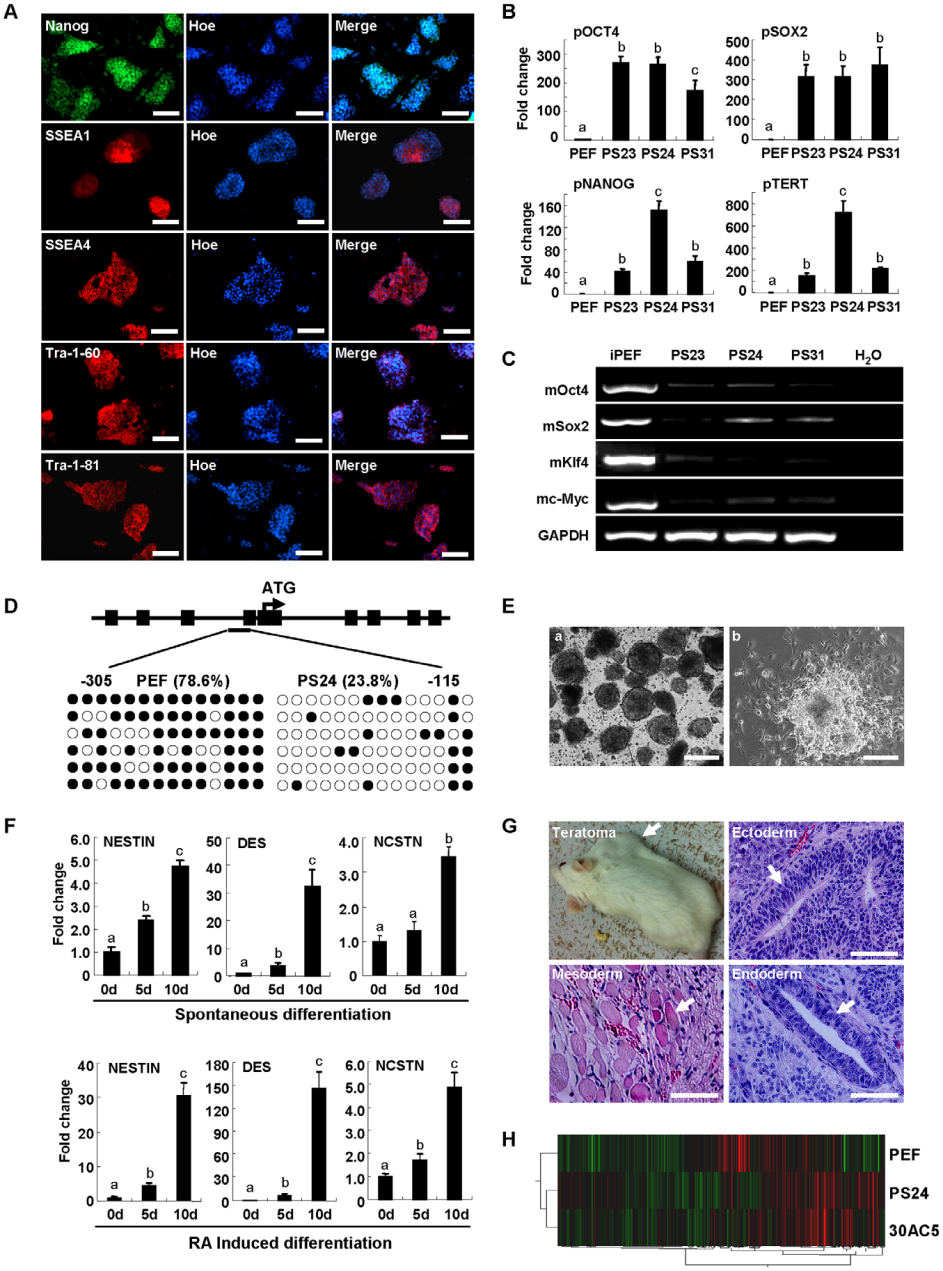
Characterization of piPS cells. A, The immunostaining of piPS cell line PS24 at passage 10. The nuclei were stained with Hoechst 33342 (Hoe). Scale bars, 100 µm. B, Quantitative RT-PCR analysis of expressions of porcine pluripotent factors (pOCT4, pSOX2, pNANOG and pTERT) in three piPS lines (PS23, PS24, PS31) and PEF. y axis represents the fold change relative to β-actin. C, Semi-quantitative RT-PCR analysis of the transgene expression (mOct4, mSox2, mKlf4, mc-Myc) in three piPS lines. iPEF, PEF cells were infected by 4F for 6 days. GAPDH was used as an internal control. D, The DNA methylation analysis of OCT4 promoter region in PS24 cell line and PEF by bisulfite sequencing. Open circles indicate unmethylated CpG, and filled circles indicate methylated CpG. E, Embryoid bodies derived from PS24 line were cultured in suspension for 5 days (a), and then cultured in tissue culture plat for the differentiation (b). Scale bars, 200 µm. F, The quantitative RT-PCR analysis of PS24 clone that was differentiate into three germ layers, NESTIN for ectoderm, DES for mesoderm and NCSTN for endoderm. The upper panels show the spontaneous differentiation for 0, 5 and 10 days, and the lower panels show the induced differentiation by RA for 0, 5 and 10 days. The y axis represents the fold change vs. GAPDH. G, Hematoxylin-eosin stained tissue section of teratoma derived from piPS clone PS24. Cells were transplanted subcutaneously in CB-17 SCID mice for 8 weeks. The teratoma and tissues representing three germ layers, neural epithelium (ectoderm), muscle (mesoderm) and gut epithelium (endoderm), are indicated by arrows. Scale bars, 100 µm. H, The heat map shows the single-linkage hierarchical clustering of microarray data (n = 20640 probes) for 2 piPS cell lines (PS24 and 30AC5) and PFF. The relative abundance of gene expression was clustered by Euclidean correlation and complete linkage. Data indicate mean ± SD (n = 3). Different letters (a, b, c) indicate significantly different between two groups, *p*<0.01 by Student’s *t* test.

More than 10 piPS cell lines were generated in this study. Three clones (PS23, PS24 and PS31) were continuously passaged for more than 30 passages (every 2–3 days for one passage) and further investigated morphologically and biochemically. Cell proliferation and colony formation were monitored by the live-cell imaging system, which showed that individual piPS cell (or small cell cluster) aggregated together to form large 3D colonies (Video S1). All three clones had the normal karyotype 38 (xy) and presented three-dimensional morphology similar to mouse ES cells and were AP positive (Fig. S2A–B). Immunofluorescence staining results demonstrated that piPS clone PS24 expressed stem cell marker NANOG, SSEA-1, SSEA-4, TRA-1-60 and TRA-1-81 (Fig. 3A). Quantitative RT–PCR analysis showed that the expression levels of endogenous pOCT4, pSOX2, pNANOG and pTERT were significantly higher in piPS clones when compared with the control PEFs (Fig. 3B). The activation of endogenous gene expression was different among the piPS cell lines. For instance, the pNANOG level in PS24 was 3–4 folds higher than that in PS23 and PS31. pTERT was also highly expressed in PS24 clone. The expression of ectopic transcription factors were greatly down regulated in three piPS cell lines, but they were not completely silenced (Fig. 3C). Meanwhile, the bisulfite genomic sequencing analysis indicated that the OCT4 promoter was unmethylated in PS24 clone (Fig. 3D). To determine the differentiation potential of piPS cells, PS24 clone was cultured in suspension to form embryoid bodies (EBs), which were then differentiated either spontaneously or in the presence of RA (Fig. 3E). The samples collected from different time points (0, 5 and 10 days) were analyzed by quantitative RT-PCR to examine the expression of differentiation markers. The results showed that markers of all three germ layers, NESTIN for ectoderm, DES for mesoderm and NCSTN for endoderm, were detected in EBs derived from PS24 clone (Fig. 3F). The PS24 cells were also injected into the CB-17 SCID mice to generate teratoma. About 8 weeks after injection, the teratoma, which contained all three germ layers of tissue stained by the histochemistry (Fig. 3G), was observed.

To further validate the piPS cells, gene expression profiles of PS24, 30AC5 cells, and PEFs were examined by Affymetrix porcine microarray to determine the degree of similarity among different piPS cell lines and PEF. 30AC5 is a partially reprogrammed piPS cell line, showing flat morphology and expressed SSEA4 and AP activity (Fig. S3). The hierarchical cluster analysis showed that PS24 and 30AC5 formed a single branch of pluripotent cell group, which was notably different from PEF (Fig. 3H), suggesting that PEF underwent extensive epigenetic reprogramming towards an ES cell-like transcriptional profiles.

### Leukemia Inhibitory Factor Suppresses the Spontaneous Differentiation of piPS Cells

**Figure 4:**
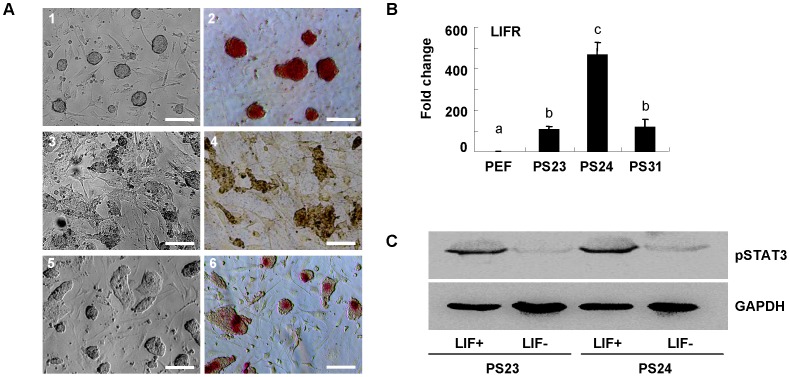
The leukemia inhibitory factor suppresses the spontaneous differentiation of piPS cells. A, The porcine PS24 cells were cultured in media with LIF and bFGF (1), without LIF (3) or without bFGF (5), respectively. The alkaline phosphatase activity was detected in above culture condition (2, 4, 6). Scale bars, 100 µm. B, The quantitative RT-PCR assay was conducted to detect the expression of LIF receptor (LIFR) in piPS cell lines. Data indicate mean ± SD (n = 3). Different letters (a, b, c) indicate significantly different between two groups, *p*<0.01 by Student’s *t* test. C, p-STAT3 protein level was investigated by Western blotting to reveal the signal pathway after the LIF treatment. GAPDH was used as an internal control.

**Figure 5:**
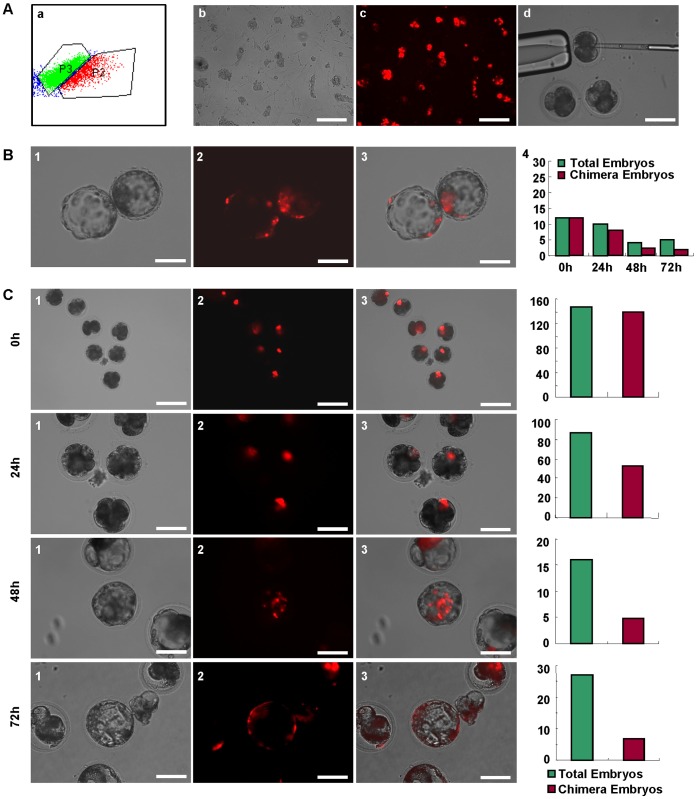
The piPS cells were used to generate chimera blastocyst in vitro. A, The PS24 cell transfected with piggyBac transposon PB[Act-RFP]DS was sorted by flow cytometer (1), and cells with RFP was fractionated in the portion 2 (P2). The PS24-RFP cells were cultured on MEF feeder (2), and showed the robust red fluorescence (3). The PS24-RFP cells were injected into pre-compacted porcine embryos through microinjection (4). Scale bars, 200 µm. B, A hatched blastocyst with the piPS cells located in both inner cell mass and trophoblast (1–3). (4) The number of chimera embryos were counted and plotted in the right panel along with the different time points, including 0 hour (0 h), 24 hours (24 h), 48 hours (48 h) and 72 hours (72 h), respectively. Scale bars, 100 µm. C, Five to ten PS24-RFP cells were delivered into pre-compacted parthenogenetic embryos by the microinjection. The chimera embryos were monitored at 0 h, 24 h, 48 h and 72 h, respectively. 1, bright field; 2, fluorescence; 3, merge. The number of total and chimera embryos was counted and plotted in the right panel. Scale bars, 100 µm in 24 h, 48 h and 72 h, and 200 µm in 0 h.

**Figure 6:**
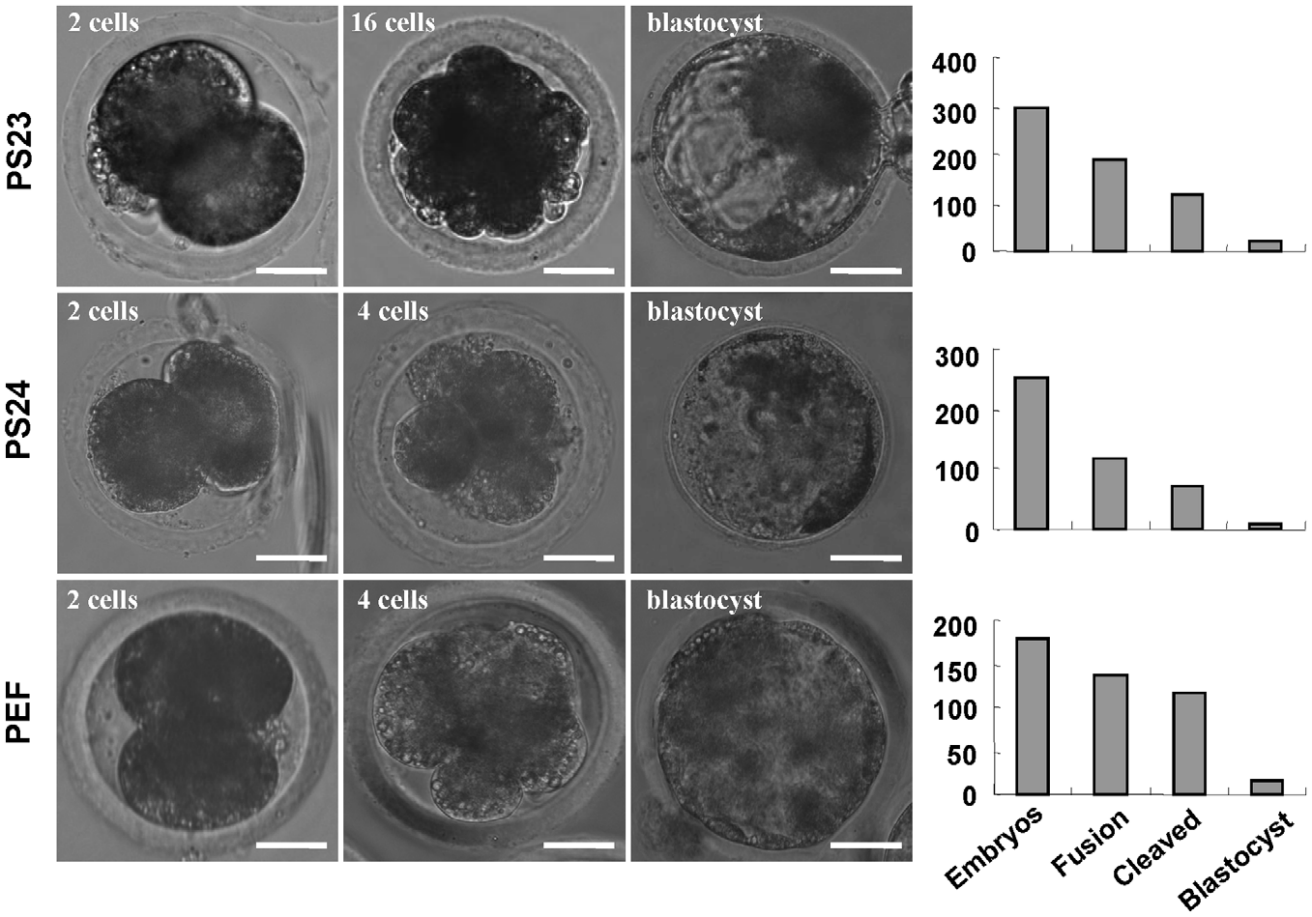
The developmental potential of NT embryos generated from piPS cells. The PS23 and PS24 lines were used as the donor cell to produce NT reconstructed embryos. The embryos at 2-cell, 4-cell and blastocyst stages were presented, and PEF cell was used as control. The number of total embryos and blastocysts were counted and plotted in the right panel. Scale bars, 25 µm.

To investigate the effect of LIF/JAK and bFGF pathway for maintaining piPS cells pluripotency, we removed LIF and bFGF from culture media. The results showed that piPS cells started to differentiate and AP activity was lost when LIF was drawn (Fig. 4A, 3, 4), but these cells could retain ES cell-like morphology and AP activity when bFGF was removed (Fig. 4A, 5, 6). Thus, LIF pathway may play a key role in maintaining pluripotency in piPS cells. The expression level of leukemia inhibitory factor receptor (LIFR) in piPS cell lines was then examined by the quantitative RT-PCR assay. The expression level of LIFR increased significantly in piPS cell lines comparing with PEFs. Among piPS clones, the LIFR level in PS24 clone was 3–4 folds higher than that in PS23 and PS31 clones (Fig. 4B). Furthermore, the Western blotting assay confirmed that the level of phosphorylated STAT3, which is a transcription factor downstream of LIF signal pathway, was significantly reduced in piPS clones upon LIF removal (Fig. 4C). In order to further confirm the role of LIF/JAK signal pathway in maintaining the pluripotency of piPS cells, JAK I inhibitor was added into medium to block the LIF/JAK signaling, and the piPS cells were loss the ES cell-likes morphology and reduced the AP activity (Fig. S4A–B), and the expression level of pluripotent genes was also significantly decreased (Fig. S4C). All these observations indicate that leukemia inhibitory factor is the key factor to maintain the self-renewal and undifferentiated state of piPS cells.

### Chimera Embryos Derived from piPS Cells

To investigate the pluripotency and developmental potential of piPS cells, PS23 and PS24 clones were used to construct chimera embryo. The piPS clones transfected by the PiggyBac transposon PB[Act-RFP]DS [33], which carried a RFP reporter, were sorted by flow cytometry. PS24-RFP cells were subcultured and used for the generation of chimeric embryos (Fig. 5A, 1–3). Initially, we used the ex vivo embryos from natural mating sows to perform microinjection (Fig. 5A, 4). In Figure 5B, a hatched blastocyst after the 3-day incubation in vitro shows that the red cells derived from piPS cells were located in both ICM and trophoblast (Fig. 5B). Five to ten PS24-RFP cells were delivered into pre-compacted 8-cell parthenogenetic embryos by the microinjection. The injected embryos were monitored at four different time points, 0, 24, 48 and 72 hours (Fig. 5C). Immediately after injection, almost all the injected embryos showed red fluorescence (93.9%). During the embryo development, the ratio of embryos carrying with PS24-RFP cells reduced from 59.8% in 24 hours, to 31.3% in 48 hours and to 25.9% in 72 hours (Fig. 5C, right panel). On the other hand, PEF-RFP cells were injected into embryos as the control, and unable to proliferate in the embryos (Fig. S5). The injected embryos derived from piPS cells could further develop into blastocysts and the blastocyst rate was similar to our previous reports [30]. The results of blastocyst generation showed the rate of chimera blastocysts from ex vivo embryos could reach up to 40%, which was higher than that from parthenogenetic embryos (Table 1). Our observations suggested that piPS cells were able to integrate into early porcine embryos and formed chimeric blastocysts.

**Table 1:** The porcine chimera blastocysts derived from piPS cells.

Cell lines	No. of embryos			Blastocysts (%)	Chimera blastocysts (%)
		Parthenogenetic	ex vivo		
PS24-RFP			12	5/12 (41.7)	2/5 (40.0)
PS24-RFP		138		27/138 (19.6)	7/27 (25.9)
PS23-RFP		74		15/74 (20.3)	4/15 (26.7)
PEF-RFP		36		6/36 (16.7 )	0 (0.00)
Ctl		41		15/41 (36.6)	n/a
Ctl			7	4/7 (57.1)	n/a

Notes: Ctl, the control treatment without injection of cells. n/a, not apply.

### NT Embryos Derived from piPS Cells

The viable live-born mice derived from mouse iPS cells by nuclear transfer or tetraploid complementation have been reported [25], [34], [35]. However, the transgenic pig based on piPS cells and nuclear transfer is still under further investigation [23], [24]. To enrich the knowledge of piPS cell’s fate, we examined the developmental potential of NT embryos derived from piPS cells. Cells of PS23 and PS24 clones were used as the donor cells to produce NT reconstructed embryos (Fig. 6). The blastocyst formation rates of PS23 and PS24 were 13.8% and 8.3%, respectively, which close to the rate of PEF control (10.7%), but the blastocyst rates after cleavage of iPS-NT embryos (21.85%) were higher than those of PEF-NT embryos (13.16%) (Table 2). This result indicates that piPS clones can be used as donor cells to produce reconstructed embryos for porcine cloning.

**Table 2:** Development of NT embryos derived from piPS and PEF cells.

Donor cells	No. of cloned embryos	No. of cleaved embryos	Blastocysts/cloned embryos (%)^1^	Blastocysts/cleaved embryos (%)
PS23	188	119	26/188 (13.83)^a^	26 (21.85)^a^
PS24	120	68	10/120 (8.33)^a^	10 (14.71)^b^
PEF	139	114	15/139 (10.79)^a^	15 (13.16)^b^

1 Different letters indicate significantly different between two groups (*p*<0.05).

## Discussion

Since the first mouse iPS cells were reported in 2006, a large number of iPS cell lines have been established in mouse, human and other animal species. Several piPS cell lines have been established [12]–[14], [23], [36], and the chimeric pigs derived from piPS cells were also reported [23]. However, most of the reported piPS cells resembled to human ES cells or mouse EpiSCs in morphology and molecular and cellular features, except for one report that showed the piPS cells in a LIF-dependent state [37]. After the comparison of culture conditions among the different piPS cell lines, we found that most of culture media were related to human iPS cell media, which the main components were KSR and bFGF [13], [14], [38]. Because of this selection condition, the obtained pig pluripotent cells resembled to human ES cell and showed the bFGF-dependence. In our studies, we compared few different cultural media for culturing porcine iPS cells, including both mouse and human ES cell culture conditions, and found that the medium with LIF and FBS could significantly increase the number of iPS clones. The observations that piPS clones express high level LIF receptors and withdrawing LIF cause spontaneous differentiation of piPS cells (Fig. 4) indicate that LIF is required to keep piPS cell self-renewal and pluripotency. On the other hand, the medium withdrawing bFGF has the minor effect on piPS clone formation (Fig 4A) [39], suggesting that bFGF may not be an essential growth factor for maintaining piPS cells. Our results agreed with the data reported by Thomson et al [40], who recently reported that LIF signaling pathway played an important role during the porcine fetal fibroblast reprogramming. Under the culture condition of FBS+ LIF, we observed the 3-D type of piPS clones in primary culture. After several passages, three piPS cell lines that retain 3-D morphology have been generated and the characteristics of pluripotency were sustained for more than 30 passages. The ES-like pluripotent cells derived from porcine ICM were LIF-dependent and showed 3-D morphology [5]. In general, our study shows that the LIF-dependent piPS cell with 3-D morphology is more closed to genuine porcine pluripotent stem cells.

The previously reported piPS cells were generated by retrovirus or lentivirus induction system [12], [13], [23], due to the efficient integration and persistent expression of transgenes by these virus vectors. However, the continued expression of exogenous reprogramming factors will likely affect the pluripotency and multi-differentiation potential of iPS cells. Therefore, the silence of transgenes is an important criterion for evaluate the cellular reprogramming, because retroviral are epigenetically silenced in pluripotent stem cells. However, the transgene silencing was not achieved in most previously reported piPS cells [12], [13]. In our studies, we monitored the expression of both exogenous and endogenous genes during the cell reprogramming, and found that, in early stage of induction (8 days), the stage-specific embryonic antigen 4 (SSEA4), a cell surface marker to identify human embryonic stem cell, was detected and co-localized with retroviral GFP^+^ cells. After two weeks induction, the ratio of SSEA4^+^/GFP**^-^** cells increased to 3.9% of total cell population. In these GFP^-^ cells, the transgenes were evidently down regulated, despite not completely silenced (Fig. 3C). This observation indicates that the induction term and culture conditions still need to be further optimized. Furthermore, the piPS cells derived from Xiao’s lab started to spontaneously differentiate soon after withdrawing doxycycline (Dox) [14], suggesting that although Dox dependent expression of four human factors initiated reprogramming process and maintained the piPS self-renewal and pluripotency, the reprogramming process had not reached to a point that endogenous pluripotent networks could uphold the pluripotency of piPS cells. Thus, identification of appropriate culture conditions for piPS is still a key issue in porcine pluripotent stem cell study.

Both less stringent tests for pluripotency, such as embryoid body and teratoma formation and differentiation in vitro [7], and more rigorous assays, including chimeras and tetraploid complementation [25], [26] have shown that mouse iPS cells are similar to the ES cells derived from ICM, which contributes to every tissue in the resulting mice, including germline tissues. Recently, Dr. Stice’s group has shown that piPS cells with human 6 factors were able to generate the chimeric offspring, demonstrating that piPS cells held a high level of plasticity [23], [24]. In this study, we detected the developmental potential of piPS cells through chimera in porcine embryos and NT to assess the piPS cells. Even though the transgenes were not fully silenced, PS23 and PS24 cells incorporated into chimera embryos and iPS-NT embryos, which developed into blastocysts. The results showed that the efficiency of piPS harboring chimera embryos was significantly higher using the fertilized embryos comparing to parthenogenetic embryos. In particularly, chimera formation in fertilized embryos was easier to develop into blastocysts (Table 1). However, the research of piPS cells is still in its infancy, and there are far away from that these cells are used as targeting cells to generate trangenic pigs for the medical research.

We have noticed that the blastocyst rate of PS24-derived NT embryos was similar to that of PEF-NT embryos. In contrast, the detailed observation revealed that the cleavage rate of iPS-NT embryos was significantly lower than that of PEF-NT embryos. Thus, the blastocyst rate of iPS-NT embryos after cleavage is higher than PEF-NT embryos (Table 2). Hochedlinger et al reported that the development of reconstructed oocytes into blastocysts was particularly sensitive to the cell-cycle stage and physical condition of the transferred nuclei [41]. In previous studies, we also found that oocyte nuclear modifications and cytoplasmic maturation underwent dramatic alterations during in vitro maturation (around 42 h) that affected subsequent embryo development [42]. The studies of the cell cycle profile of mouse iPS cells cultured with LIF showed that LIF-induced DNA synthesis caused the high percentage of iPS cells in S and G2 phases [43]. Therefore, we speculate that the large proportion of piPS cells in the S and G2/M phases influences the rate of cleavage division of iPS-NT embryos. On the other hand, the PEF cells in the G_0_ phase can obtain the higher cleavage rate. Thus, the blastocyst rate of piPS-NT embryos was higher than that of PEF-NT embryos based on the cleavage.

In conclusion, we successfully established several piPS cell lines based on the induction by mouse OSKM transcriptional factors under the culture condition with FBS, LIF and bFGF. The piPS clones preserve the morphological and biological features of self-renewal and pluripotency as the mouse ES and iPS cells. The generation of chimeric embryos and NT embryos derived from piPS clones is a practical means to examine the quality of iPS cells ex vivo.

## Supporting Information

Figure S1 **Analysis of the silence of transgenes during the reprogramming.** A, PEFs were infected by retroviruses containing GFP, the proportion of infected cells is >90%. B, The changes of cell morphology during the reprogramming. Small colonies with GFP fluorescence appeared at 7–8 days after infection (a–b). 10–12 days after infection, some colonies showed absent GFP fluorescence (c–d), and these colonies could grow and double the size when continuously cultured for 3–5 days (e–f). Scale bars, 200 µm. C. The semi-quantitative RT-PCR assay was conducted to detect the expression of transgenes (mOct4, mSox2, mKlf4, mc-Myc) and GFP in the GFP^-^ colonies iF1 and iF2, and GAPDH was used as an internal control.
(TIF)

Figure S2 **A, Three-dimensional morphology of piPS cell lines (PS23.** PS24 and PS31) are similar to the morphology of mouse ES cells. The alkaline phosphatase activities are positive (right panel). Scale bars, 50 µm. B, The karyotype of three piPS cell lines (PS23, PS24 and PS31), which shows 38 (xy). Scale bars, 25 µm.
(TIF)

Figure S3 **The piPS cell line 30AC5 shows the flat morphology, alkaline phosphatase activity and expression of SSEA4.** The nuclei were stained with Hoechst 33342 (Hoe). Scale bars, 200 µm.
(TIF)

Figure S4 **The piPS cell line PS23 was cultured in medium with JAK I inhibitor for 5 days.** A, the morphology of PS23 cells after JAK I inhibitor treatment. B, Alkaline phosphatase assay of PS23 cells after the treatment. Scale bars, 200 µm. C, The expression of *OCT4*, *SOX2* and *NANOG* in PS23 cells that were treated by JAK I inhibitor or removed LIF for 5 days. Data indicate mean ± SD (n = 3). Different letters (a, b, c) indicate significantly different between two groups, *p*<0.01 by Student’s *t* test.
(TIF)

Figure S5 **The porcine blastocysts derived from the parthenogenetic embryos that were injected with PEF-RFP cells in the pre-compact 8-cell stage.** Scale bars, 200 µm in A–B, 100 µm in C–D.
(TIF)

Table S1 **Features of piPS cells derived from different laboratories.**
(DOC)

Table S2 **Name and sequence of primers used in this study.**
(DOC)

Video S1 **The video of live-cell imaging of piPS clones formation.** Porcine PS24 cells were treated with 0.05% Trypsin, and then seeded on a 35 mm culture plate for 24 hours. The plate was then transferred to the live-cell imaging system. Cell growth was instantly monitored and recorded in every 2 min by Leica microscope (Leica AF6000, Germany). The video was then formed by the imaging system. Scale bars, 50 µm.
(AVI)
