# Supplementary figures and images for: Porcine Induced Pluripotent Stem Cells Require LIF and Maintain Their Developmental Potential in Early Stage of Embryos
Source: PLoS One. 2012 Dec 14;7(12):e51778. doi: 10.1371/journal.pone.0051778 (PMC3522612; doi:10.1371/journal.pone.0051778)

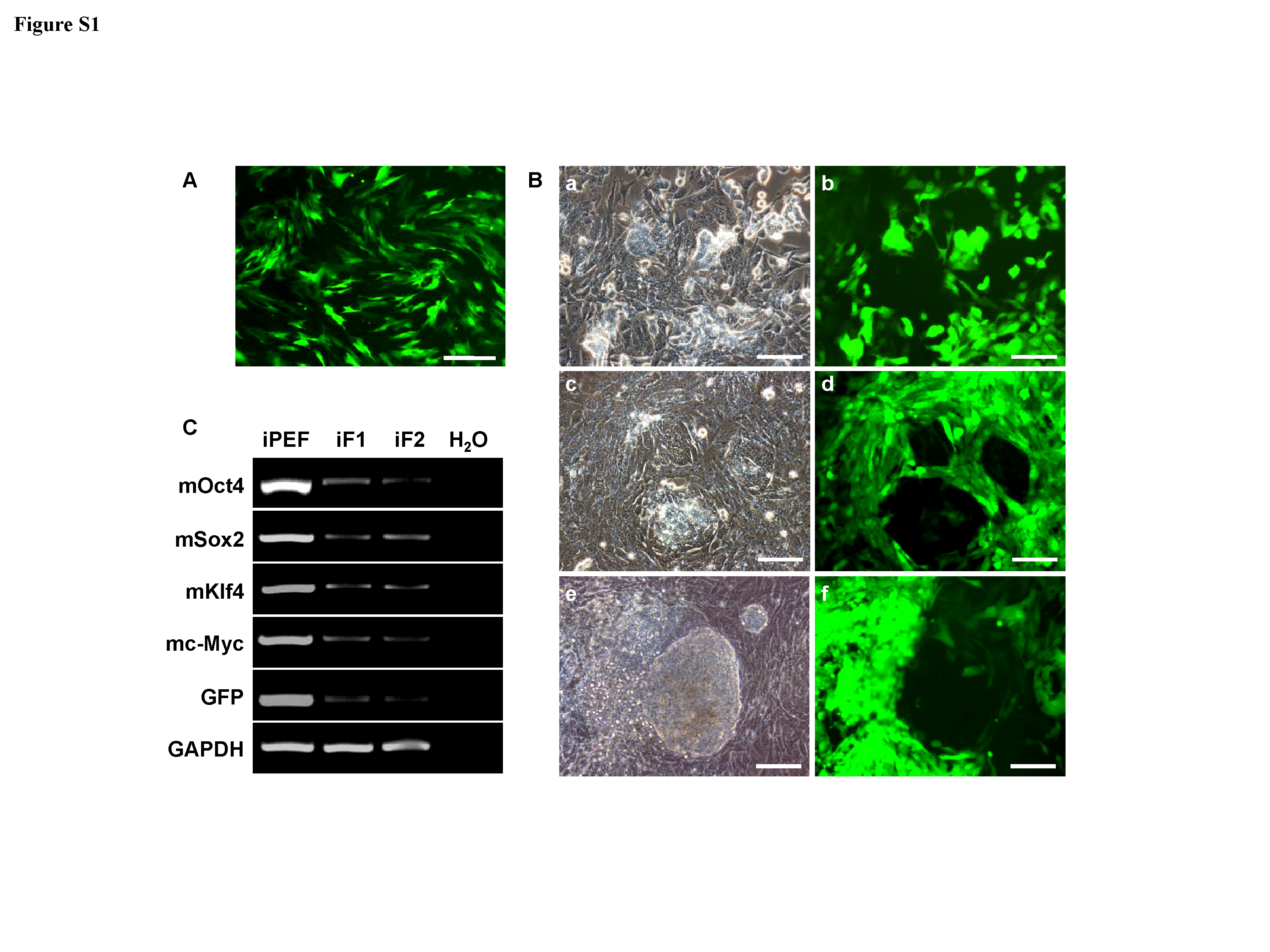

Supplement: Figure S1 — Analysis of the silence of transgenes during the reprogramming. A, PEFs were infected by retroviruses containing GFP, the proportion of infected cells is >90%. B, The changes of cell morphology during the reprogramming. Small colonies with GFP fluorescence appeared at 7–8 days after infection (a–b). 10–12 days after infection, some colonies showed absent GFP fluorescence (c–d), and these colonies could grow and double the size when continuously cultured for 3–5 days (e–f). Scale bars, 200 µm. C. The semi-quantitative RT-PCR assay was conducted to detect the expression of transgenes (mOct4, mSox2, mKlf4, mc-Myc) and GFP in the GFP- colonies iF1 and iF2, and GAPDH was used as an internal control. (TIF) [file pone.0051778.s001.tif]

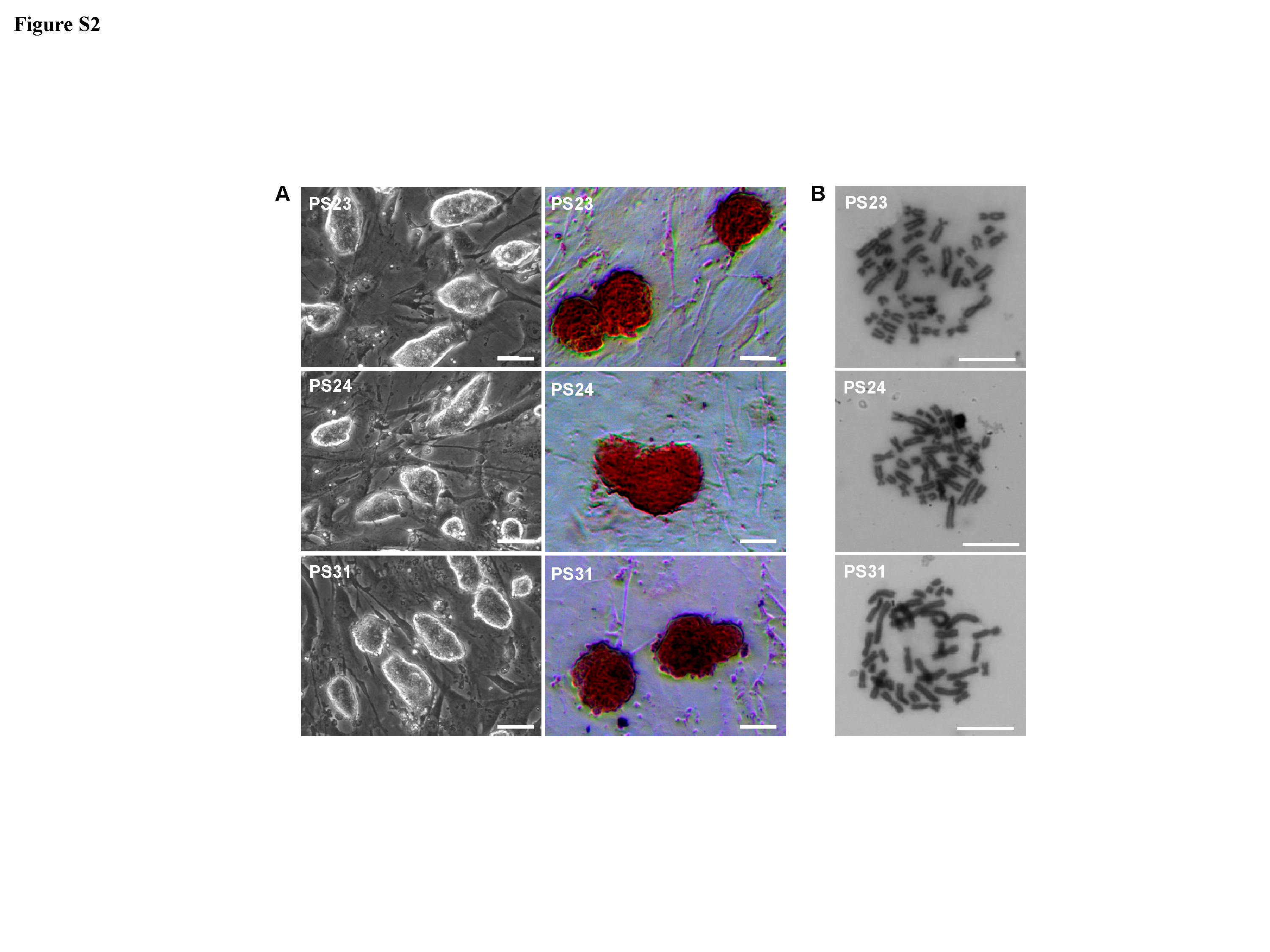

Supplement: Figure S2 — A, Three-dimensional morphology of piPS cell lines (PS23. PS24 and PS31) are similar to the morphology of mouse ES cells. The alkaline phosphatase activities are positive (right panel). Scale bars, 50 µm. B, The karyotype of three piPS cell lines (PS23, PS24 and PS31), which shows 38 (xy). Scale bars, 25 µm. (TIF) [file pone.0051778.s002.tif]

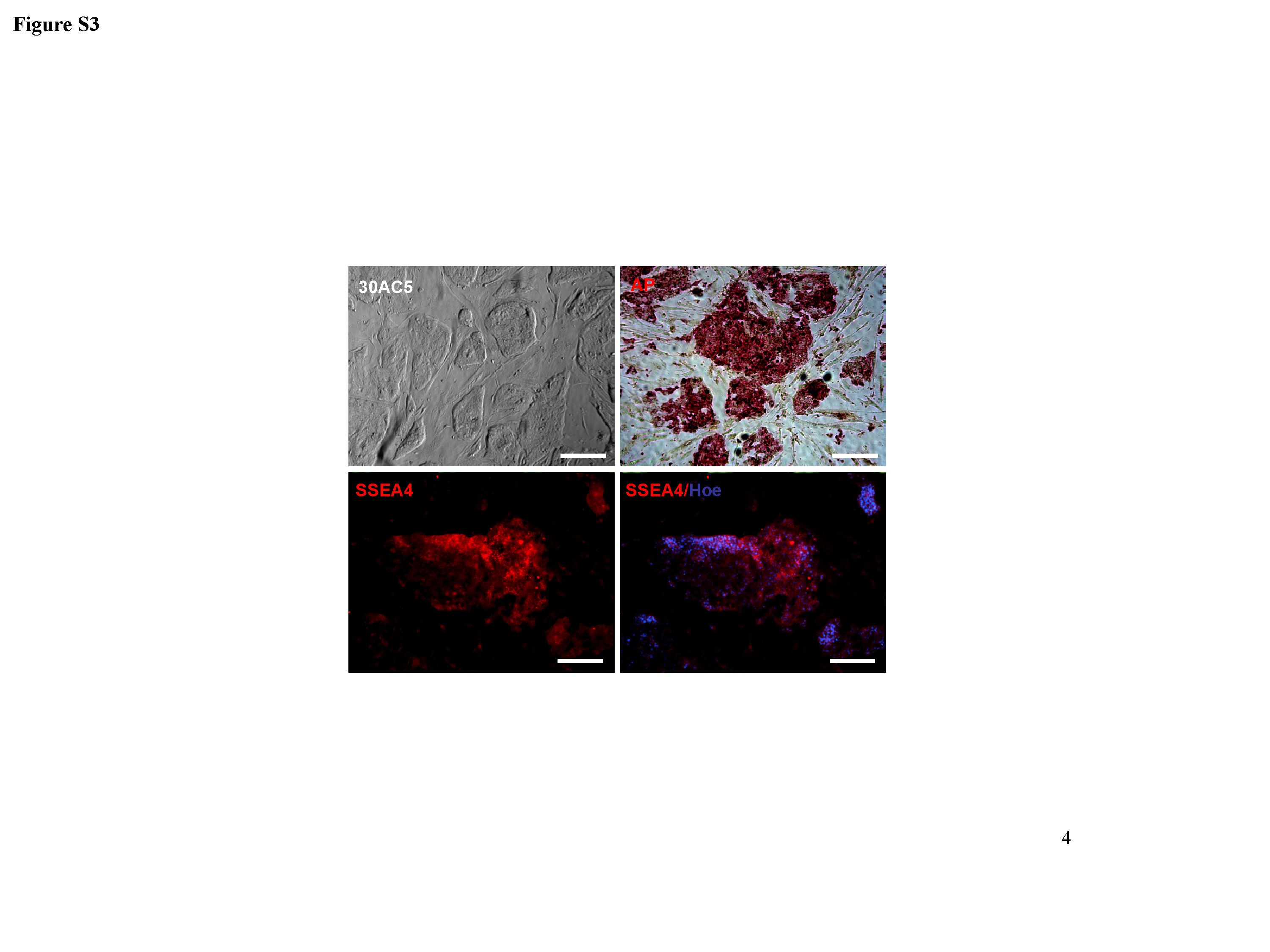

Supplement: Figure S3 — The piPS cell line 30AC5 shows the flat morphology, alkaline phosphatase activity and expression of SSEA4. The nuclei were stained with Hoechst 33342 (Hoe). Scale bars, 200 µm. (TIF) [file pone.0051778.s003.tif]

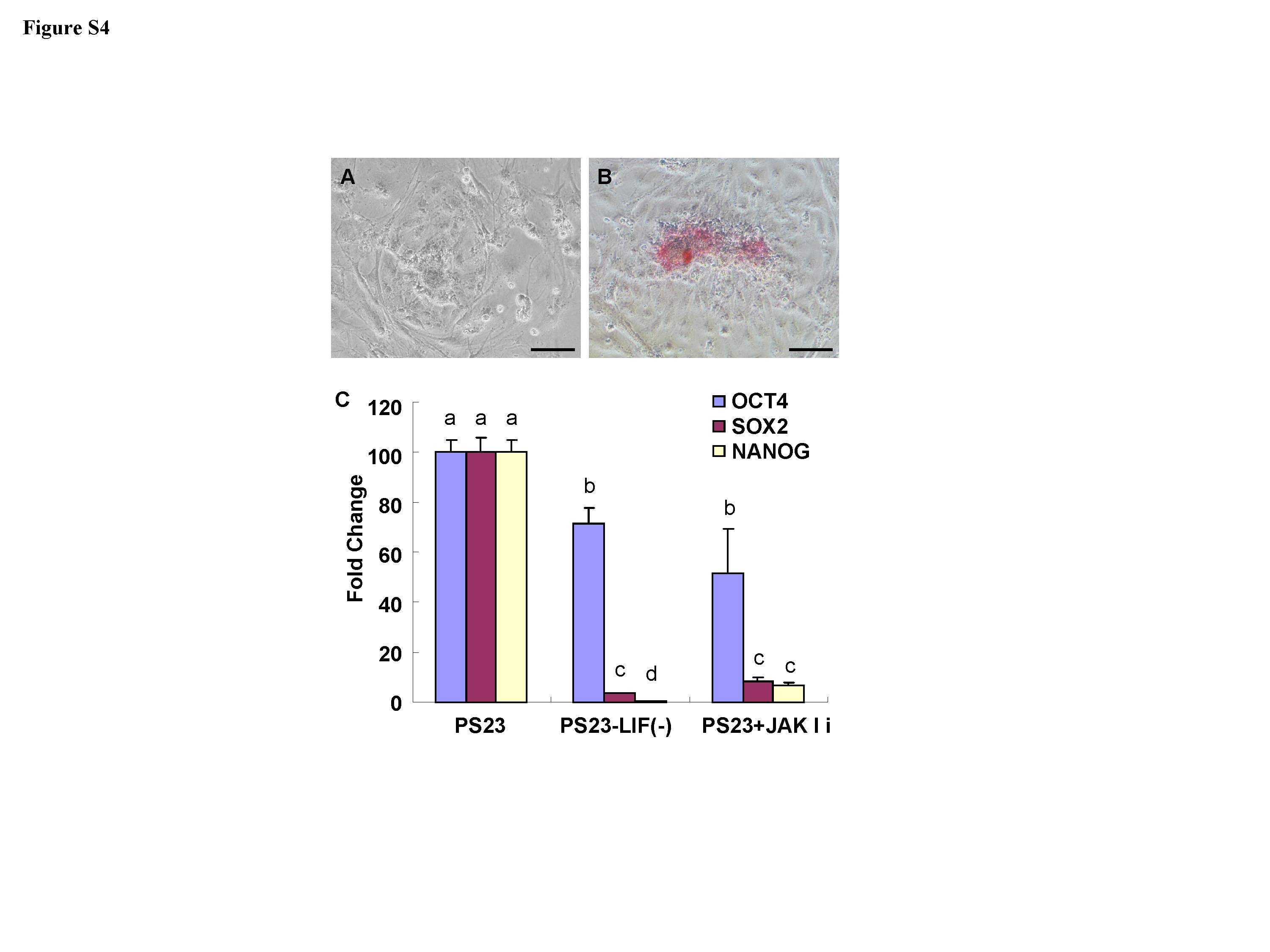

Supplement: Figure S4 — The piPS cell line PS23 was cultured in medium with JAK I inhibitor for 5 days. A, the morphology of PS23 cells after JAK I inhibitor treatment. B, Alkaline phosphatase assay of PS23 cells after the treatment. Scale bars, 200 µm. C, The expression of OCT4, SOX2 and NANOG in PS23 cells that were treated by JAK I inhibitor or removed LIF for 5 days. Data indicate mean ± SD (n = 3). Different letters (a, b, c) indicate significantly different between two groups, p<0.01 by Student’s t test. (TIF) [file pone.0051778.s004.tif]

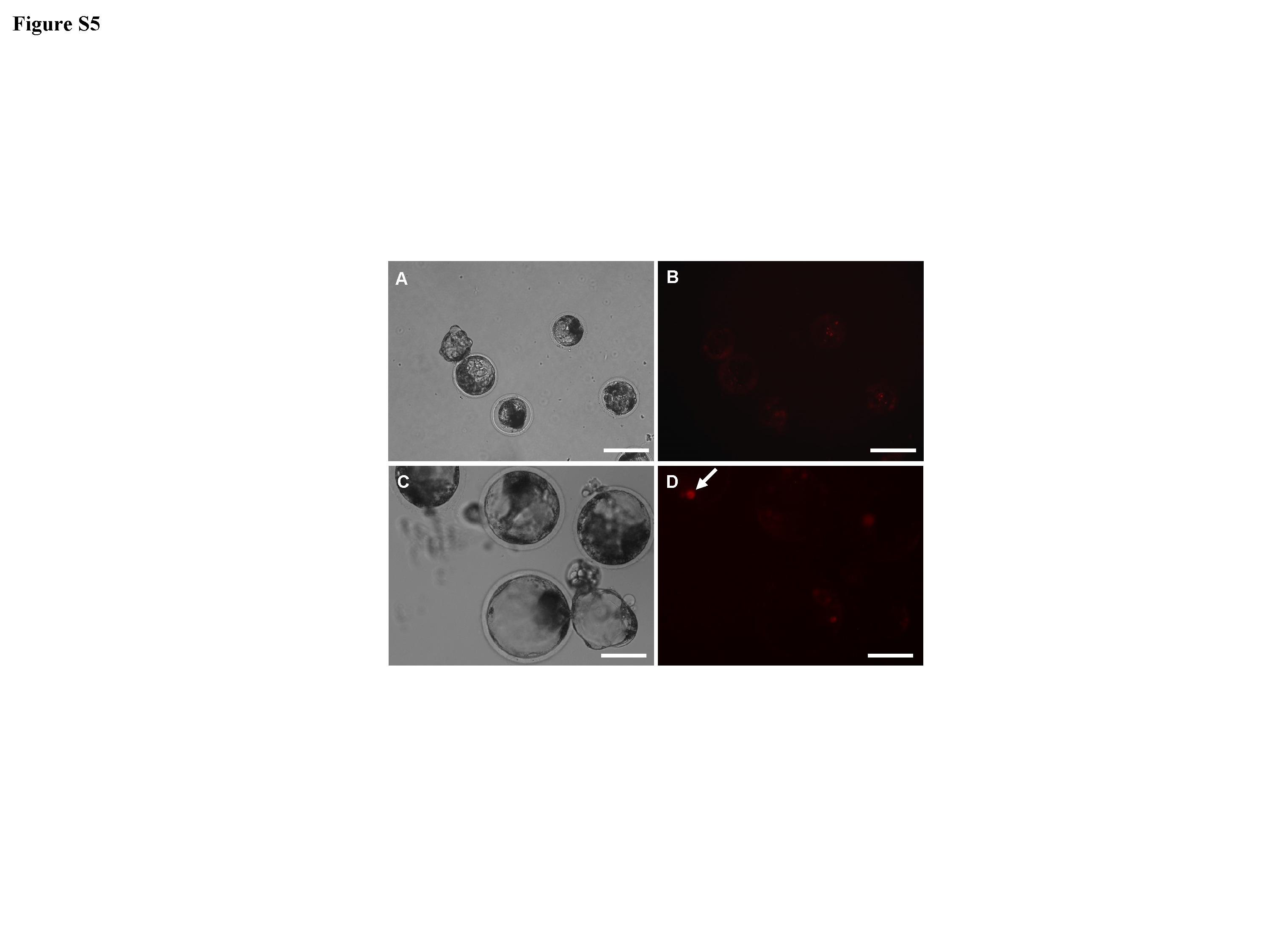

Supplement: Figure S5 — The porcine blastocysts derived from the parthenogenetic embryos that were injected with PEF-RFP cells in the pre-compact 8-cell stage. Scale bars, 200 µm in A–B, 100 µm in C–D. (TIF) [file pone.0051778.s005.tif]
