# Supplementary material for: Porcine Induced Pluripotent Stem Cells Require LIF and Maintain Their Developmental Potential in Early Stage of Embryos
Source: PLoS One. 2012 Dec 14;7(12):e51778. doi: 10.1371/journal.pone.0051778 (PMC3522612; doi:10.1371/journal.pone.0051778)
Supplement: Table S1 — Features of piPS cells derived from different laboratories. (DOC) [file pone.0051778.s006.doc]

**Table S1. Features of piPS cells derived from different laboratories**

| Feature | Mouse iPS cells | Human iPS cells | piPS cells from Esteban | piPS cells from Wu | piPS cells from Ezashi | piPS cells from this study |
| --- | --- | --- | --- | --- | --- | --- |
| Factors | Mouse 4F | Human 4F | Mouse 4F | Human 4F | Human 4F | Mouse 4F |
| Morphology | 3-D | Flat | Flat | Flat | Flat | 3-D |
| MEF feeder | + | + | + | + | + | + |
| SSEA1 | + | - | N/A | N/A | + | + |
| SSEA3 | - | + | N/A | + | - | N/A |
| SSEA4 | - | + | + | + | - | + |
| TRA-1-60 | - | + | N/A | + | - | + |
| TRA-1-81 | - | + | N/A | + | - | + |
| Cytokine | LIF | bFGF | bFGF | - | bFGF | LIF |

+, Positive; -, Negative; N/A, not available
