# Supplementary material for: Porcine Induced Pluripotent Stem Cells Require LIF and Maintain Their Developmental Potential in Early Stage of Embryos
Source: PLoS One. 2012 Dec 14;7(12):e51778. doi: 10.1371/journal.pone.0051778 (PMC3522612; doi:10.1371/journal.pone.0051778)
Supplement: Table S2 — Name and sequence of primers used in this study. (DOC) [file pone.0051778.s007.doc]

**Table S2. Name and sequence of primers used in this study**

Primer DNA Sequence Size (bp) Accession No.

mOct4 S:5’-TGTTCCCGTCACTGCTCTG 461 NM_013633

mSox2 S:5’-GGCCATTAACGGCACACTG 298 NM_011443

mKlf4 S:5’-CGATGAACTGACCAGGCACTA 437 NM_010637

mMyc S:5’-TTCAAGCAGACGAGCACAAG 392 AK087961

pMXs-R A:5’-GGCGTTACTTAAGCTAGCTTG

GFP S:5’-GACGGGAACTACAAGACACG 318

A:3’-CGAAAGGGCAGATTGTGTGG

pOCT4 S:5’-CAAACTGAGGTGCCTGCCCTTC 190 CT737281.12

A:5’-ATTGAACTTCACCTTCCCTCCAACC

pSOX2 S:5’-GTTCCATGGGCTCAGTGGTCAAG 347 CU914271.8

A:5’-AAGCGTACCGGGTTTTTCTCCATAC

pNANOG S:5’-TCACCAATGCCTGAGGTTTATG 149 DQ447201

A:5’-GGGCTTGTGGAAGAATCAGG

pTERT S:5’-AAGACGCTGTTTGCTGTGC 99 AY785158.1

A:5’-CCGCCACGCCCTGTGGATGT

LIFR S:5’-CCCAAATAATGTTGAGGTTCTG 118 SSU91518

A:5’-GTTTTCGGGTTCTGCATCAG

DES S:5’-CCTCAACTTCCGAGAAACAAGC 108 AF136188.1

A:5’-TCACTGACGACCTCCCCATC

NCSTN S:5’-CAGCAAAGAACTGGAGTTCATCACTCT 159 XM_001928786.1

A:5’-AGGAAAAGCTGGGGTCCTCTTCAG

NESTIN S:5’-TAGAGCCCGTGTTGGAAGAT 408 XM_001925549

A:5’-CATCACTTCCACTGTGGTGC

Oct4-Me S:5’-GAAGAGGGGTTTAATATTTGGTTTT 281 CT737281.12

A:5’-CCACCCACTAACCTTAACCTCTAA

β-ACTIN S:5’-TGCTCGCCAACGTTTACA 153 EU655628

A:5’-CAAGCCGGAGGAAAAATG

GAPDH S:5’-ACCTGCCGCCTGGAGAAACC 252 M_001206359

A:5’-GACCATGAGGTCCACCACCCTG
